# GLP-1 receptor agonists in patients with cancer are associated with reduced all-cause mortality and hospitalization

**DOI:** 10.1210/clinem/dgaf703

**Published:** 2026-01-02

**Authors:** Aditya Mahadevan, Aidan Vosooghi, Jagmeet S Arora, Ruthvik Sunil Kumar, Gagandeep Singh, Katy K Tsai, Zoe Quandt

**Affiliations:** Department of Medicine, University of California, San Francisco, San Francisco, CA 94143, USA; Department of Medicine, University of Michigan, Ann Arbor, MI 48109, USA; School of Medicine, University of California, Irvine, CA 92617, USA; Department of Medicine, University of Kentucky, Lexington, KY 40506, USA; Department of Computer Science, University of California, Davis, CA 95616, USA; Division of Hematology/Oncology, Department of Medicine, University of California, San Francisco, San Francisco, CA 94143, USA; Division of Endocrinology, Department of Medicine, University of California, San Francisco, San Francisco, CA 94143, USA; Diabetes Center, University of California, San Francisco, San Francisco, CA 94143, USA

**Keywords:** GLP-1RA, cancer, survival, tirzepatide, semaglutide, liraglutide, exenatide, dulaglutide

## Abstract

**Context:**

Glucagon-like peptide-1 receptor agonists (GLP-1RAs) have been reported to decrease cancer incidence, but less is known about their potential in patients with active cancer. Preclinical studies have demonstrated that GLP-1RAs inhibit progression of solid tumor malignancies via downregulation of cellular proliferation pathways and improved glycemic control. Despite these promising findings, studies characterizing the effects of GLP-1RAs in patients with active cancer are limited.

**Objective:**

To evaluate the effects of GLP-1RAs on mortality and hospitalization in patients with type 2 diabetes and active cancer compared to those receiving metformin.

**Methods:**

Using TriNetX, a global database comprising more than 120 million patients, we identified an overall cohort of 3747 patients with type 2 diabetes who received GLP-1RAs within 3 months of starting systemic therapy and identified 52 061 patients receiving metformin in the same timeframe as a control cohort. Additional subanalyses stratified patients by glycated hemoglobin A_1c_ (HbA_1c_) range, obesity, and by participants “newly started” on their first instance of GLP-1 RA within 3 months of starting cancer treatment.

**Results:**

Patients receiving GLP-1RAs had significantly reduced mortality both in the overall monotherapy setting (hazard ratio [HR]: 0.875; 95% CI, 0.778-0.985; *P* = .0268) and the new-start setting (HR: 0.786; 95% CI, 0.662-0.934; *P* = .0062) cohorts. Secondary analyses found lower rates of all-cause hospitalization, sepsis, major adverse cardiovascular events, pulmonary embolism, and pneumonia in patients on GLP-1RAs. Subanalyses stratified by body mass index and HbA_1c_ did not meet statistical significance.

**Conclusion:**

Patients with diabetes and cancer who received GLP-1RAs experienced superior survival outcomes and reduced rates of hospitalization compared to patients receiving metformin. Additionally, patients already on metformin and newly started on GLP-1RAs demonstrated superior survival outcomes compared to patients newly started on insulin. Further prospective, well-controlled studies are needed to evaluate the benefits of GLP-1RAs in patients with diabetes and cancer.

Since US Food and Drug Administration approval of semaglutide in 2017, the use of glucagon-like peptide-1 receptor agonists (GLP-1RAs) has dramatically increased in patients living with type 2 diabetes mellitus (T2D) (1). GLP-1RAs mimic GLP-1, a peptide produced by pancreatic islet cells to enhance insulin release while suppressing glucagon, thereby promoting improved glycemic control (2-4). In patients with cancer, a disease that relies on glucose metabolism to accelerate tumorigenesis, glycemic control is of particular interest (5). To this end, patients with cancer and T2D with optimized glycemic control may have improved survival outcomes compared to patients with poorer glycemic control (6, 7). Additionally, alternative antihyperglycemic medications such as metformin may also be associated with improved outcomes in patients with T2D who develop cancer (8-11).

While the positive effects of metformin in patients with T2D and cancer are relatively well established, studies evaluating the effects of GLP-1RAs on cancer outcomes are limited. Thus far, GLP-1RA usage has been associated with a reduced risk of certain cancers, though these outcomes are similar to those of patients receiving metformin (12). Additionally, preclinical studies of in vitro human models and in vivo mice models of liver, colorectal, and pancreatic cancer have demonstrated that GLP-1RAs inhibit tumor progression via several potential mechanisms, including downregulation of cellular proliferation pathways, as well as glycemic control itself (13-15). In patients with active cancer, Chiang and colleagues demonstrated that patients receiving GLP-1RAs and concomitant immunotherapy experience fewer cardiovascular adverse events, in line with a prior meta-analysis demonstrating significant major adverse cardiovascular events (MACE) reduction in patients with and without T2D who received GLP-1RAs (16, 17). Additionally, a recent retrospective study found that patients diagnosed with neuroendocrine tumors who received GLP-1RAs experienced lower rates of all-cause mortality (18). This may be explained by downregulation of insulin-like growth factors, which are expressed at higher rates in neuroendocrine tumors (18-21).

However, data comparing head-to-head outcomes in patients with cancer receiving metformin monotherapy vs GLP-1RA monotherapy stratified by glycemic control have not been performed to date. To our knowledge, we performed the first comprehensive analysis investigating associations between continued or new-start GLP-1RA and survival in patients with T2D diagnosed with solid and hematologic malignancies across various ranges of glycemic control.

## Materials and methods

### Cohort selection

We used TriNetX, a global collaborative network comprising more than 120 million patients, to identify cohorts of individuals with T2D by International Classification of Diseases 10 (ICD-10) code (E11), who were diagnosed with any of the top 10 causes of cancer-related death in men and women, including the following cancers: malignant neoplasms of digestive organs (C15-C26), malignant melanoma of skin (C43), malignant neoplasms of the urinary tract (C64-C68), malignant neoplasm of the breast (C50), malignant neoplasms of lymphoid, hematopoietic, and related tissue (C81-C96), malignant neoplasms of the uterine corpus (C54), malignant neoplasms of the ovary (C56), and malignant neoplasms of the brain (C71) (22). To account for differences in cancer treatment modalities over time, analyses were time-limited to include only patients from January 1, 2010, onward.

### Comparator groups

For the “overall cohort,” patients who received any instance of GLP-1RA up to 3 months before and/or after systemic cancer therapy were compared to patients who received any instance of metformin up to 3 months before and/or after systemic therapy. For the “new-start” cohort, patients previously on metformin who received their first instance of GLP-1RA up to 3 months before and/or after systemic cancer therapy were compared to patients previously on metformin who received their first instance of insulin up to 3 months before and/or after systemic therapy.

### Exposure to glucagon-like peptide-1 receptor agonists

Medication start was defined as the first instance of a medication denoted by a Prescription for Electronic Drug Information Exchange (RxNorm) within the TriNetX database. For the GLP-1RA overall cohort, GLP-1RA exposure was defined as any instance of receiving a GLP-1RA (liraglutide, dulaglutide, semaglutide, exenatide, tirzepatide) within 3 months before or after first systemic cancer therapy administration. To account for GLP-1RA and metformin coprescription, patients receiving metformin up to 3 months before first systemic cancer therapy administration and onward were excluded from the GLP-1RA cohort. In the “new-start” cohorts, GLP-1RA exposure was defined as the first instance of receiving a GLP-1RA up to 3 months before and/or after systemic therapy. Similarly, insulin exposure was defined as the first instance of receiving insulin up to 3 months before and/or after systemic therapy.

### Outcomes

The primary outcome of interest was survival, an outcome explicitly recorded by TriNetX via extraction from electronic health records, and hazard ratios (HRs) with 95% CIs were calculated. Death was directly extracted from electronic health records via TriNetX. Secondary outcomes were all-cause hospitalization and each leading cause of cancer-related or diabetes-related hospitalization (23, 24). These included MACE, diabetic ketoacidosis (DKA), hyperosmolar hyperglycemic state (HHS), pneumonia, sepsis, and pulmonary embolism (PE) (24). Risk ratios (RRs) with 95% CIs were calculated for secondary outcomes.

### Definitions

Systemic cancer therapy comprised alkylating agents, anthracyclines, antimetabolites, immune checkpoint inhibitors (ICIs), targeted monoclonal antibodies, platinum-based chemotherapy, and tyrosine kinase inhibitors (Supplementary Table S1) (25). “Overall” cohorts were defined as receiving any instance of either GLP-1RA or insulin up to 3 months before and/or after the first administration of systemic therapy. “New-start” cohorts were defined as receiving the first instance of either GLP-1 RA or insulin within 3 months before or after the first administration of systemic therapy. “Optimal glycemic control” was defined as glycated hemoglobin A_1c_ (HbA_1c_) values between 6.5% and 7%. “Moderate glycemic control” was defined as HbA_1c_ values between 7% and 9%. “Suboptimal glycemic control” was defined as HbA_1c_ values greater than 9%. HbA_1c_ values were measured within 3 months of systemic cancer therapy administration.

### Statistical analysis

The index event for all analyses was the first day of administration of systemic anticancer therapy. Prior to analyses, cohorts were 1:1 propensity score–matched, controlling for age, sex, race, ethnicity, type of malignancy, presence of distant metastases, surgery, radiation, systemic therapy, and key comorbidities including hyperlipidemia, hypertension, smoking, chronic kidney disease, and obesity.

Kaplan-Meier survival analyses were conducted within the TriNetX platform to compare survival outcomes between cohorts with log-rank tests defining significance. Kaplan-Meier survival analyses were started 6 months after systemic cancer therapy and at least 3 months after any instance of GLP-1RA or metformin. This process, referred to as “landmarking,” was conducted to minimize effects of the immortal time bias between the cohorts. The Benjamini-Hochberg procedure was used to adjust for multiple comparisons.

Forest plots and flow charts were generated using R (R version 4.3.3) to display HRs, RRs, and 95% CIs for primary and secondary outcomes.

### Subanalysis and sensitivity analysis

Additional subanalyses were conducted between these two cohorts, stratified by glycemic control based on HbA_1c_ up to 3 months before and/or after systemic therapy start and with/without obesity (denoted by ICD-10 code E66).

## Results

**Figure 1.**
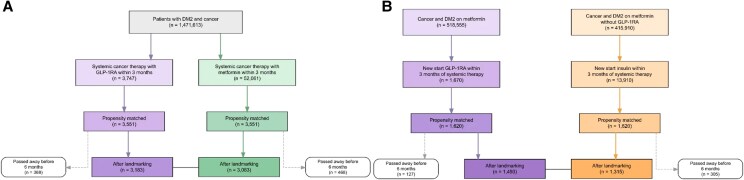
Cohort flow diagram for overall and new start cohorts. Flow diagrams for propensity-matched cohorts. A, Glucagon-like peptide-1 receptor agonist (GLP-1RA) monotherapy vs metformin monotherapy comparison showing propensity matching after 6-month landmarking. B, GLP-1RA new start vs insulin new start comparison showing propensity matching after 6-month landmarking.

Our study identified 3747 patients with T2D who received a GLP-1RA within 3 months of starting systemic therapy (Table 1) without a concurrent metformin prescription (“GLP-1RA monotherapy”). Within the control cohort, we identified 52 061 patients with T2D who received metformin within 3 months of starting systemic therapy (Fig. 1) without a concurrent GLP-1RA prescription. After propensity score matching, participants were evenly distributed by age, sex, cancer type, and most comorbidities. After propensity matching was performed, 3551 patients were compared from each cohort (see Table 1). The GLP-1RA cohort had a lower proportion of White patients (72.8% vs 75.0%; *P* = .0375) and a higher proportion of Black patients (15.5% vs 14.1%; *P* = .0883). For analyses comparing “new-start” GLP-1RA, 1670 patients with T2D were identified who received their first instance of GLP-1RA up to 3 months before or after starting systemic therapy (see Fig. 1).

**Table 1.** Overall cohort demographic table

Baseline characteristic	GLP-1RA monotherapy	Metformin monotherapy	*P*
Total No. of patients	3551	3551	
Age			
Mean (SD), y	65.3 (10.4)	65.3 (10.8)	.7711
Sex, n (%)			
Female	1989 (56.0%)	1986 (55.9%)	.9428
Male	1561 (44.0%)	1565 (44.1%)	.9238
Race/Ethnicity, n (%)			
White	2585 (72.8%)	2662 (75.0%)	.0375
Black or African American	551 (15.5%)	500 (14.1%)	.0883
Hispanic or Latino	217 (6.1%)	218 (6.1%)	.9605
Asian	116 (3.3%)	103 (2.9%)	.3722
Comorbidities, n (%)			
Essential (primary) hypertension	2952 (83.1%)	2983 (84.0%)	.3209
Hyperlipidemia, unspecified	2511 (70.7%)	2534 (71.4%)	.5474
Overweight and obesity	2200 (62.0%)	2177 (61.3%)	.5746
CKD	1263 (35.6%)	1209 (34.0%)	.1786
Nicotine use	697 (19.6%)	699 (19.7%)	.9524
Cancer diagnoses, n (%)			
Malignant neoplasms of digestive organs	995 (28.0%)	1019 (28.7%)	.5275
Malignant neoplasms of breast (C50)	663 (18.7%)	674 (19.0%)	.7385
Malignant neoplasms of lymphoid, hematopoietic and related tissue	567 (16.0%)	591 (16.6%)	.4408
Malignant neoplasms of respiratory and intrathoracic organs	505 (14.2%)	478 (13.5%)	.3535
Melanoma and other malignant neoplasms of skin	489 (13.8%)	502 (14.1%)	.6562
Secondary malignant neoplasm of other and unspecified sites	473 (13.3%)	438 (12.3%)	.2142
Malignant neoplasms of urinary tract	457 (12.9%)	439 (12.4%)	.5200
Malignant neoplasm of kidney, except renal pelvis	264 (7.4%)	241 (6.8%)	.2883
Malignant neoplasm of corpus uteri	224 (6.3%)	203 (5.7%)	.2945
Malignant neoplasm of bladder	202 (5.7%)	208 (5.9%)	.7602
Malignant neoplasms of male genital organs	152 (4.3%)	136 (3.8%)	.3358
Malignant neoplasm of prostate	142 (4.0%)	123 (3.5%)	.2342
Malignant neoplasm of ovary	131 (3.7%)	135 (3.8%)	.8026
Malignant neoplasm of cervix uteri	44 (1.2%)	38 (1.1%)	.5051
Malignant neoplasm of brain	38 (1.1%)	36 (1.0%)	.8152
Malignant neoplasm of renal pelvis	34 (1.0%)	22 (0.6%)	.1074
Treatment/Procedures, n (%)			
Surgery	3238 (91.2%)	3275 (92.2%)	.1114
Radiation	550 (15.5%)	525 (14.8%)	.4078

Abbreviations: CKD, chronic kidney disease; GLP-1RA, glucagon-like peptide-1 receptor agonist.

**Figure 2.**
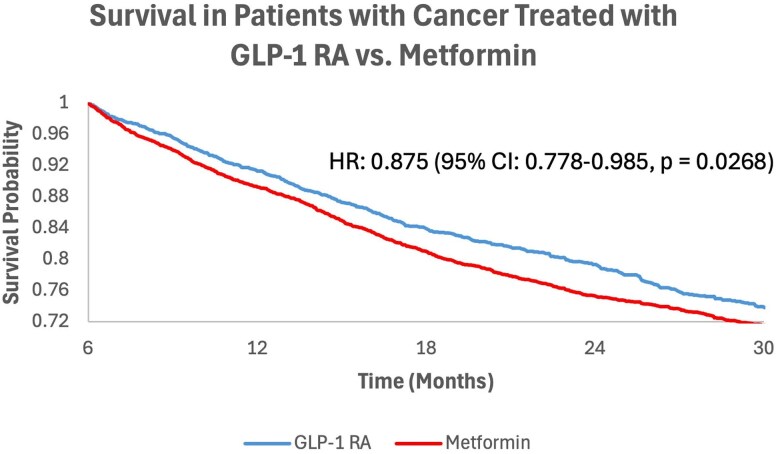
Overall survival in patients on glucagon-like peptide-1 receptor agonist (GLP-1RA) monotherapy as compared to metformin monotherapy. Kaplan-Meier survival curves depicting overall survival from 6 months to 2.5 years post cancer treatment for the GLP-1RA overall vs metformin overall cohorts.

Patients with T2D who received GLP-1RA monotherapy within 3 months of starting systemic therapy (Fig. 2) had significantly reduced all-cause mortality compared to patients receiving metformin monotherapy within 3 months of systemic therapy (HR: 0.875; 95% CI, 0.778-0.985; *P* = .0268).

**Figure 3.**
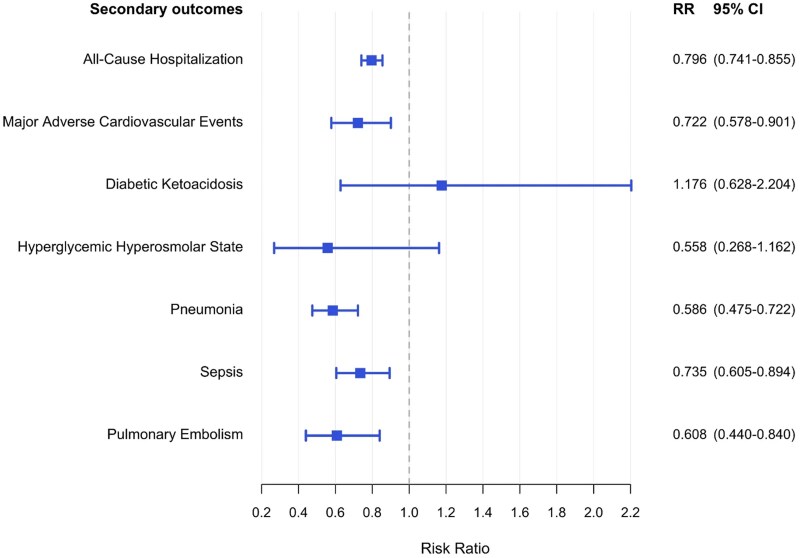
Hospitalization-specific outcomes in glucagon-like peptide-1 receptor agonist (GLP-1RA) monotherapy as compared to metformin monotherapy. Forest plot depicting all-cause hospitalization and leading causes of cancer and diabetes-related hospitalization in patients on GLP-1RA monotherapy and metformin monotherapy.

Additionally, patients with T2D who received GLP-1RA monotherapy within 3 months of starting systemic cancer therapy demonstrated significantly reduced all-cause hospitalization compared to patients receiving metformin monotherapy (RR: 0.796; 95% CI, 0.741-.855; *P* = .0004) (Fig. 3). Patients exposed to GLP-1RA experienced significantly reduced rates of pneumonia (RR: 0.586; 95% CI, 0.475-0.722; *P* = .0004) and sepsis (RR: 0.735; 95% CI, 0.605-0.894; *P* = .004) (see Fig. 3). Notably, rates of MACE (RR: 0.722; 95% CI, 0.578-0.901; *P* = .0053) and PE (RR: 0.608; 95% CI, 0.44-0.84; *P* = .004) were significantly lower in the GLP-1RA cohort. Rates of DKA and HHS did not differ significantly between the GLP-1RA and metformin cohorts (see Fig. 3).

**Figure 4.**
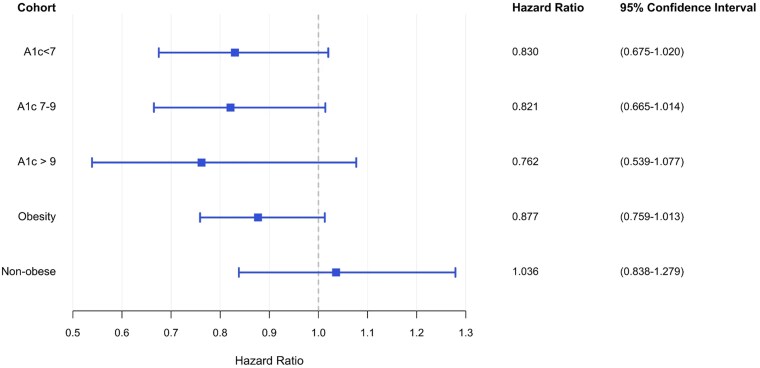
Glucagon-like peptide-1 receptor agonist monotherapy vs metformin monotherapy associations stratified by hemoglobin A_1c_ and body mass index (BMI). Forest plot depicting survival outcomes in subanalyses stratifying by hemoglobin A_1c_ and BMI.

When stratifying by HbA_1c_, survival outcomes between GLP-1RA monotherapy and metformin monotherapy approached but did not meet statistical significance. Across the optimal (HR: 0.83; 95% CI, 0.675-1.02; *P* = .128), moderate (HR: 0.821; 95% CI, 0.665-1.014; *P* = .128), and suboptimal diabetes control (HR: 0.762; 95% CI, 0.539-1.077; *P* = .128) (Fig. 4). When stratifying by body mass index (BMI), obese patients demonstrated improved survival that approached statistical significance (HR: 0.877; 95% CI, 0.759-1.013; *P* = .156), while nonobese patients demonstrated comparable survival (HR: 1.036; 95% CI, 0.838-1.279; *P* = .746).

**Figure 5.**
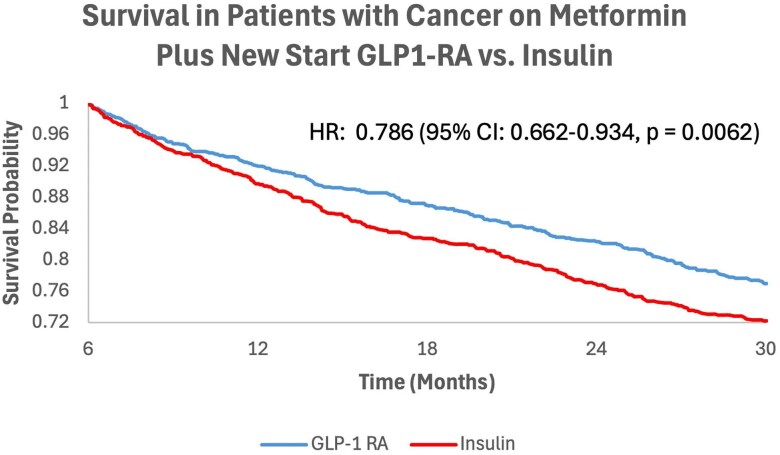
Addition of glucagon-like peptide-1 receptor agonist (GLP-1RA) vs insulin to prior medications in association with survival. Kaplan-Meier survival curves depicting overall survival from 6 months to 2.5 years post cancer treatment for the GLP-1RA “new start” vs insulin “new start” cohorts.

For the “new-start” insulin cohort, 13 910 patients with T2D were identified who received their first instance of insulin up to 3 months before and/or after starting systemic therapy. After propensity matching, 1620 patients were compared from each “new-start” cohort (see Fig. 1). Compared to patients on metformin newly starting insulin for T2D, patients on metformin newly starting GLP-1RA for T2D experienced significantly lower rates of mortality (HR: 0.786; 95% CI, 0.662-0.934; *P* = .0062) (Fig. 5).

## Discussion

To our knowledge, we performed the first comprehensive analysis comparing outcomes of patients with cancer and T2D who received GLP-1RAs vs metformin across the leading causes of cancer-related death in patients both with solid tumor and hematologic malignancies, in multiple treatment settings and across various ranges of BMI and glycemic control. Patients receiving GLP-1RA monotherapy had significantly reduced mortality and all-cause hospitalization in the overall cohort compared to patients receiving metformin monotherapy. Additionally, patients receiving GLP-1RA experienced significantly lower levels of sepsis, pneumonia, PE, and MACE compared to patients receiving metformin. When stratifying by HbA_1c_ and obesity, cohort-based survival differences in GLP-1RA monotherapy and metformin monotherapy did not meet statistical significance.

Patients receiving GLP-1RA monotherapy experienced improved survival compared to patients receiving metformin monotherapy, adding to the small body of literature examining GLP-1RA in patients with active cancer. To date, studies examining the effects of patients receiving GLP-1RAs with active cancer are limited. Analyses of tumor molecular profiling have found that GLP-1R expression is associated with variable prognoses and GLP-1 signaling-related genes may be reduced in cancer cells (26, 27). A study of patients with T2D receiving ICIs found that GLP-1RA was associated with reduced all-cause mortality compared to dipeptidyl peptidase 4 inhibitors (DPP-4is) (16). Our findings are in stark contrast to a prior study of women with gynecologic cancers, in which a pooled group of “second-tier” antihyperglycemics, including GLP-1RAs, demonstrated inferior survival outcomes to patients receiving metformin (28). This discrepancy may be in part explained by the fact that the pooled comparator group also included other antihyperglycemics, such as DPP-4is, for which GLP-1RAs have shown superior outcomes in patients with cancer (16). Given that GLP-1R expression may be correlated with poorer outcomes in gynecologic malignancies, it follows that our analyses, which incorporate a variety of hematologic and solid tumor malignancies, differ from prior literature (26).

Additionally, in patients already on metformin, the addition of “new-start” GLP-1RA demonstrated significantly improved survival compared to patients newly started on insulin. While insulin is sometimes used for metformin-refractory, suboptimally controlled diabetes, insulin has been associated with poorer survival outcomes in patients with active cancer (29, 30). These survival trends extend to studies comparing insulin usage to noninsulin antihyperglycemics (31). These findings are supported by preclinical studies demonstrating that insulin upregulates cellular proliferation pathways implicated in cancer, activates the insulin receptor and insulin-like growth factor receptor implicated in tumorigenesis, and may even enhance tumor angiogenesis (32-34). The superior survival outcomes in patients receiving GLP-1RAs compared to insulin suggest that GLP-1RAs are viable antihyperglycemics in patients with cancer, particularly when paired with metformin. This is particularly relevant as patients with suboptimally controlled diabetes may require multiple antihyperglycemics to achieve optimal glycemic control. Despite close propensity matching, access to medications may differ between these populations and should be considered when interpreting these findings.

When stratifying by HbA_1c_, patients receiving GLP-1RAs in the optimal, moderate, and suboptimal glycemic control groups demonstrated trends toward improved survival that did not meet statistical significance. This may be in part attributed to reduced power, as the sample sizes of these subanalyses varied from 11% to 28% of the size of the primary analysis. While these trends suggest that GLP-1RAs may have beneficial effects across a wide range of HbA_1c_ levels, further prospective, highly powered studies are needed to further investigate these findings and verify that the survival associations are not mediated by glycemic control or obesity.

In comparing the obesity and nonobesity subanalyses, patients with obesity demonstrated a trend toward improved survival that did not meet statistical significance. This trend was not seen in the nonobese cohort. GLP-1RAs are known for causing weight loss via multiple mechanisms, including inhibition of gastric emptying and appetite suppression (35, 36). In patients with active cancer, cancer-related cachexia also leads to weight loss and anorexia via release of inflammatory cytokines (37, 38). The role of obesity and cancer is complex and multidirectional, and studies conflict regarding the role of overweight and obesity as a protective factor in patients with active cancer (39, 40). While we controlled for obesity as part of our propensity matching in the primary cohort, cohort-based differences in the obesity and nonobese cohorts suggest that patients with cancer and obesity may be more likely to benefit from GLP-1RA therapy. Additionally, it is unlikely that someone who has cancer-related cachexia or anorexia would be continued on or newly started on a GLP-1RA, underscoring the need for prospective data. Taken together, these findings indicate the need for prospective studies to further explore the interplay of GLP-1RA and obesity in patient outcomes.

Beyond mortality benefit, patients receiving GLP-1RAs also demonstrated a mild though statistically significant reduction in all-cause hospitalization compared to patients receiving metformin. While GLP-1RAs have previously been shown to increase the risk of pancreatitis-related hospitalizations, they also may carry a lower risk of all-cause hospitalization compared to DPP-4is (41-43). However, to our knowledge, no study has evaluated the effects of GLP-1RA on all-cause hospitalization in patients with cancer. Reduced rates of hospitalization in patients receiving GLP-1RA may be in part attributed to reduced rates of adverse outcomes that lead to hospitalization, as indicated via the secondary outcomes we investigated. Among the common causes of cancer-related hospitalization we investigated, patients receiving GLP-1RAs experienced lower rates of pneumonia, sepsis, PE, and MACE.

GLP-1RA users experienced lower rates of pneumonia and sepsis, adding to the growing body of literature expounding on these benefits. Recent literature has found that patients receiving GLP-1RAs may experience lower rates of pneumonia and severe sepsis compared to those on metformin and similar rates compared to patients receiving sodium-glucose cotransporter 2 inhibitors (SGLT2is) (44, 45). While the exact mechanisms mediating these benefits are unclear, GLP-1RAs have been shown to reduce rates of acute lung injury in pneumonia-induced sepsis via regulation of surfactant proteins in mice (46). Given that pneumonia and sepsis are among the leading causes of hospitalization in patients with cancer, further investigation is needed to better understand the role of GLP-1RAs in potentially mitigating these adverse outcomes (23).

Additionally, we found that patients receiving GLP-1RAs experienced lower rates of PE. Preclinical studies suggest that GLP-1RAs may reduce platelet aggregation via several mechanisms involving cyclic adenosine monophosphate (cAMP) and nitric oxide production, which support a theoretical risk reduction in venous thromboembolism (VTE) (47-49). However, our findings differ from prior meta-analyses that found similar or increased risk of VTE compared to placebo (50, 51). This could be in part due to differences in treatment population, as the aforementioned analyses did not exclusively include patients with cancer. Additionally, despite matching for key medical comorbidities, we were unable to assess performance status via the TriNetX database, which may play a role in development of VTE in patients with cancer (52). Given the dramatic effects of cancer-related VTE on patient survival, further investigation is needed to understand the relationship between GLP-1RAs and cancer-related VTE (53).

Prior studies have demonstrated significantly reduced rates of MACE, including cardiovascular death, from initiation of GLP-1RAs in patients with T2D (54, 55). In alignment with these studies, we found similar rates of MACE in patients receiving GLP-1RAs vs metformin. Our findings contrast with a post hoc analysis of the Harmony Outcomes trial, in which the addition of liraglutide compared to placebo in baseline metformin users did not lead to a reduction in the primary outcome of composite cardiovascular death, myocardial infarction, or stroke (56). However, studies of MACE reduction from GLP-1RAs specifically in patients undergoing treatment for cancer are limited. A recent study of patients with cancer treated with ICIs found that those treated with GLP-1RAs experienced significantly lower incidence of MACE compared to patients receiving non–GLP-1RA antihyperglycemic agents. Preclinical studies have suggested that GLP-1RAs may decrease cardiovascular risk via an antiatherosclerotic effect mediated by attenuating atherosclerotic plaque development and decreasing circulating levels of inflammatory cytokines, including tumor necrosis factor, interferon γ, and osteopontin (57). Prospective studies are needed to better understand the effect of GLP-1 RA on MACE risk in patients undergoing treatment for cancer, and whether these risks are attenuated by use of other antihyperglycemic agents.

While HHS occurred at similar rates to patients receiving metformin, DKA occurred at higher rates in patients receiving GLP-1RAs that did not meet statistical significance. While a signal exists linking DKA and GLP-1RAs, retrospective studies comparing rates of DKA in metformin, DPP-4is, SGLT2is, and GLP-1RAs found that GLP-1RAs had lower risk of DKA compared to SGLT2is (58, 59). No prior studies to date have examined the relationship between DKA and GLP-1RA usage in patients with active cancer. Similarly, the link between HHS and GLP-1RA is poorly understood with no documented associations in the literature to date. Further research is needed to understand the relationship between GLP-1RA exposure and DKA/HHS in patients with cancer.

This study had a number of limitations. These include reliance on retrospective data, potential inaccuracies related to ICD-10 coding, absence of true staging data, nuances of systemic therapy, inability to assess medication adherence, and absence of cause-specific mortality. To account for inaccuracies in ICD-10 coding, we carefully chose codes used in prior studies in these topic areas. To avoid reliance on billing codes to capture diabetes, we used HbA_1c_ to verify the presence of diabetes and account for differences in glycemic control. Additionally, though we were unable to fully control for stage or tumor characteristics including tumor grade, we controlled for the presence of distant metastases (ICD-10 code C79) as a surrogate for metastatic disease, in addition to controlling carefully for treatment modality and propensity matching for key variables including age, sex, race, cancer type, surgery, radiation, and medical comorbidities. While differences in systemic therapies were captured by propensity matching, we were unable to determine if these agents were used with palliative or curative intent, or in the adjuvant vs neoadjuvant setting. Additionally, while intervals were carefully chosen to reflect GLP-1RA and metformin administration, due to electronic medical record–based limitations, we were unable to explicitly confirm medication adherence, despite the important role this plays in patient outcomes. Within the diabetes data, the TriNetX database cannot capture whether patients actually take their prescribed medications. There can also be other clinical conditions not fully captured by our clinical variables that lead to physician medication choice and prescription of GLP-1RAs that are associated with survival (confounding by indication). Finally, while we were unable to account for cause-specific mortality, we carefully chose secondary outcomes that reflect substantial sources of morbidity and mortality in patients with cancer.

Given the role of optimal glycemic control in patients with diabetes and cancer, evaluating the efficacy of new treatments is of the utmost importance. Compared to standard-of-care metformin, we found that patients exposed to GLP-1RAs exhibited significantly reduced mortality and all-case hospitalization, irrespective of start time. Additionally, new-start GLP-1RAs in patients on metformin exhibited improved survival compared to patients started on insulin, suggesting that GLP-1RAs may be a viable second-line agent in active cancer. Additionally, the observed reduced rates of hospitalization, severe infection, and atherosclerotic and thrombotic events suggest further unexplored benefits of GLP-1RAs. Future prospective studies should evaluate the benefits of GLP-1RAs in patients with cancer in comparison to other antihyperglycemic medications to further elucidate these potential benefits.

## Data Availability

The data from this study are available via the TriNetX platform. Restrictions apply to the availability of this data, which were used under license for this study. Data are available from the authors with the permission of TriNetX.

## Abbreviations

BMI: body mass index
DKA: diabetic ketoacidosis
DPP-4is: dipeptidyl peptidase 4 inhibitors
GLP-1RA: glucagon-like peptide-1 receptor agonist
HbA_1c_: glycated hemoglobin A_1c_
HR: hazard ratio
HHS: hyperosmolar hyperglycemic state
ICD-10: International Classification of Diseases 10
ICI: immune checkpoint inhibitor
MACE: major adverse cardiovascular events
PE: pulmonary embolism
RR: risk ratio
SGLT2is: sodium-glucose cotransporter 2 inhibitors
T2D: type 2 diabetes mellitus
VTE: venous thromboembolism
